# Prevalence and co-existence of cardiometabolic risk factors and associations with nutrition-related and socioeconomic indicators in a national sample of Gambian women

**DOI:** 10.1038/s41598-021-91592-7

**Published:** 2021-06-08

**Authors:** Nicolai Petry, Fabian Rohner, Modou Cheyassin Phall, Bakary Jallow, Abdou Aziz Ceesay, Yankuba Sawo, Momodou K. Darboe, Samba Barrow, Aminatta Sarr, Pa Ousman Ceesay, Malang N. Fofana, Andrew M. Prentice, Rita Wegmüller, James P. Wirth

**Affiliations:** 1 GroundWork, 7306 Fläsch, Switzerland; 2 National Nutrition Agency, Banjul, The Gambia; 3 UNICEF, Banjul, The Gambia; 4 Medical Research Council Unit the Gambia at London, School of Hygiene and Tropical Medicine, Atlantic Boulevard, Fajara, Banjul, The Gambia; 5 Gambia Bureau of Statistics, Banjul, The Gambia

**Keywords:** Cardiovascular biology, Risk factors

## Abstract

Cardiovascular diseases (CVD) are on the rise in Sub-Saharan Africa, and a large proportion of the adult population is thought to suffer from at least one cardiometabolic risk factor. This study assessed cardiometabolic risk factors and the contribution of nutrition-related indicators in Gambian women. The prevalence and co-existence of diabetes (elevated glycated hemoglobin (HbA1c ≥ 6.5%) or prediabetes (HbA1c ≥ 5.7% to < 6.5%), hypertension (systolic blood pressure ≥ 140 mmHg or diastolic blood pressure ≥ 90 mmHg), obesity (body mass index (BMI) ≥ 30.0) and inflammation (C-reactive protein (CRP) > 3 mg/L or alpha-1-acid glycoprotein (AGP) > 1 g/L) and the contribution of nutrition related and socioeconomic indicators were measured in non-pregnant women 15–49 years of age in the Gambia using data from a nationally representative cross-sectional stratified survey. Nationally, 54.5% (95% CI: 47.4, 61.4) of 1407 women had elevated HbA1c. Of these, 14.9% were diabetic and 85.1% were prediabetic. Moreover, 20.8% (95% CI 17.8, 20.0) of 1685 women had hypertension, 11.1% (95% CI 9.0, 13.7) of 1651 were obese and 17.2% (95% CI 5.1, 19.6) of 1401 had inflammation. At least one of the aforementioned cardiometabolic risk factor was present in 68.3% (95% CI 63.0, 73.1) of women. Obesity increased the risk of hypertension (aRR 1.84; 95% CI 1.40, 2.41), diabetes (aRR 1.91; 95% CI 1.29, 2.84), elevated HbA1c (aRR 1.31; 95% CI 1.14, 1.51) and inflammation (aRR 3.47; 95% CI 2.61, 4.61). Inflammation increased the risk of hypertension (aRR 1.42; 95% CI 1.14, 1.78). Aging increased the risk of hypertension, obesity and inflammation. Further, inadequate sanitation increased the risk for diabetes (aRR 1.65; 95% CI 1.17, 2.34) and iron deficiency increased the risk of elevated HbA1c (aRR 1.21; 95% CI 1.09, 1.33). The high prevalence of cardiometabolic risk factors and their co-existence in Gambian women is concerning. Although controlling obesity seems to be key, multifaceted strategies to tackle the risk factors separately are warranted to reduce the prevalence or minimize the risk of CVD.

## Introduction

More than half of the yearly global deaths can be attributed to non-communicable diseases (NCDs)^1^ and between 1990 and 2017, the total number of disability adjusted life years (DALYs) lost due to NCDs increased by 67% and the contribution of NCDs to the total burden of disease in Sub-Saharan Africa (SSA) increased by 60%. For the majority of NCDs, this drastic increase is solely attributable to the growing and aging population. However, this is not true for diabetes mellitus, certain cancers and certain cardiovascular diseases (CVD), which exhibit age-standardized increasing rates^2^ and account for large parts of NCD attributable deaths^1^. The World Health Organization has defined 9 voluntary targets in prevention and control of NCDs to be attained by 2025, including the reduction in mortality from CVD, diabetes, cancer, and chronic respiratory diseases and a halt in the rise in the prevalence of diabetes and obesity^3^. NCDs are alarmingly on the rise in SSA, mainly driven by an increasing incidence of cardiovascular risk factors^4^. SSA currently experiences an epidemiological transition, which is characterized by an increase in the prevalence of NCDs^2^ and a persistently-high incidence of the main infectious diseases, such as malaria^5^, tuberculosis^6^, and HIV^7^. It is estimated that NCDs will have superseded communicable diseases as the major cause of death by 2030^4^. To date, the majority of NCDs in SSA are CVDs, but data on NCDs and their risk factors in SSA countries are scarce, rendering a comprehensive assessment difficult, albeit indispensable for the development of evidence-based policies and the implementation of health programs^4^. Globally, hypertension, diabetes, obesity and inflammation are major cardiometabolic risk factors^8,9^ and a risk factor assessment of NCDs has shown that most adults in SSA are exposed to at least one risk factor^2^. Hypertension is mainly caused by excessive consumption of alcohol, smoking, physical inactivity, unhealthy diet, overweight and obesity, and high salt intake^1^, whereas diabetes mellitus type 2 is mainly driven by overweight and obesity^10,11^. In 2019, globally, 463 million people were diabetic, 19 million of whom were in SSA. Projections estimate that these numbers will increase, so that by 2045 about 700 million and 47 million people will suffer from diabetes globally and in SSA, respectively^12^. Moreover, inflammation is recognized as a critical cardiometabolic risk factor for CVD, particularly for atherosclerosis^13^. As NCDs are posited to cause morbidity and mortality in The Gambia^14^, a current and thorough investigation of their prevalence and associated risk factors is warranted. This study thus assessed the prevalence, intra-individual co-existence and determinants of established cardiometabolic risk factors, such as diabetes mellitus, pre-diabetes, hypertension, inflammation, and obesity in a national sample of non-pregnant women as part of The Gambia National Micronutrient Survey 2018^15^.

## Results

**Figure 1:**
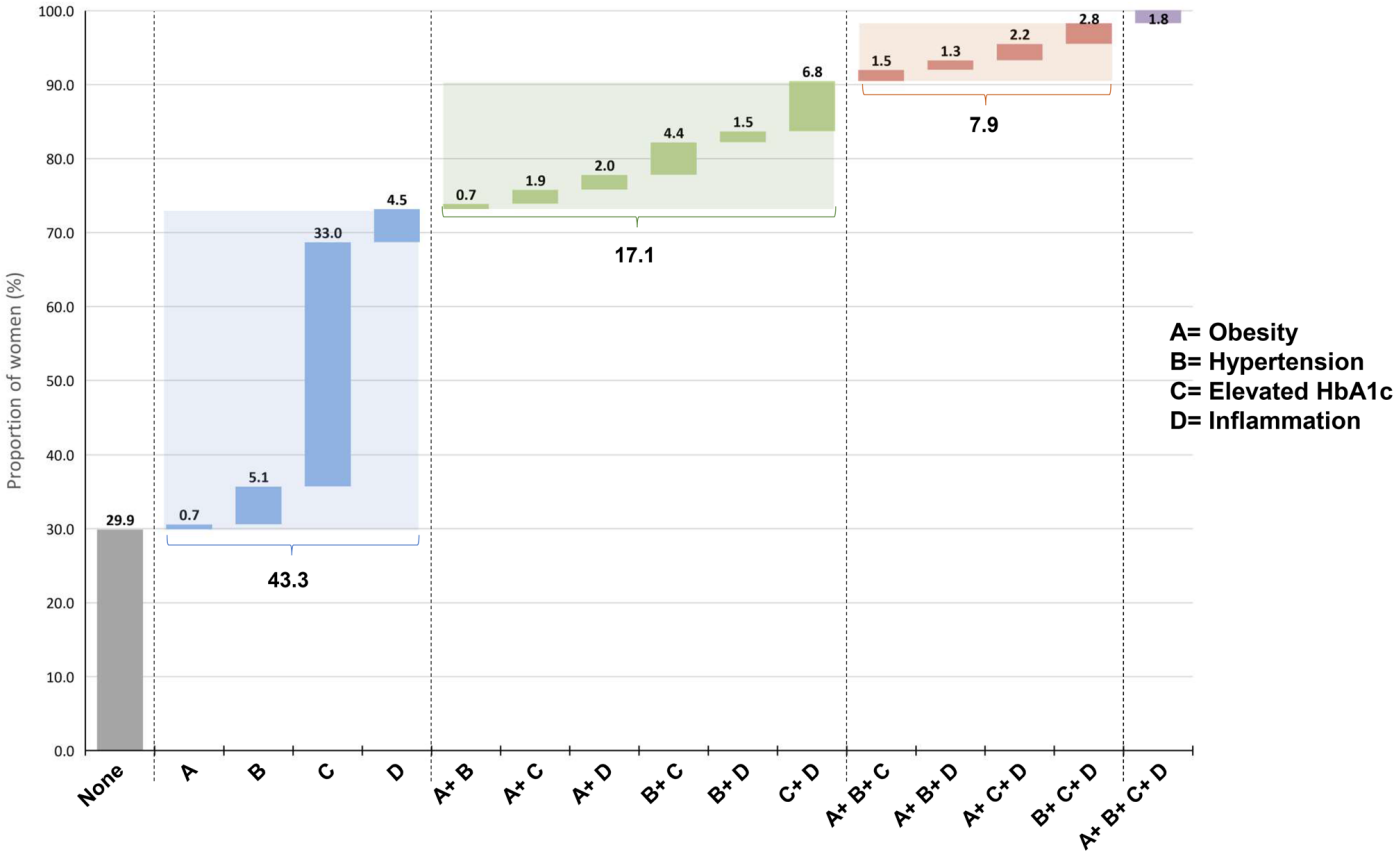
Coexistence of obesity (BMI ≥ 30.0), hypertension (systolic ≥ 140 mmHg or diastolic ≥ 90 mmHg), elevated HbA1c (≥ 5.7%) and inflammation (CRP > 3 mg/L and/ or AGP > 1 g/L) in the same woman (includes only the 1391 women with data on all cardio metabolic risk factors); grey = proportion of women without risk factor, blue = proportion of women with one risk factor, green = proportion of women with two risk factors, orange = proportion of women with three risk factors, purple = proportion of women with four risk factors.

The study sample comprised 1703 non-pregnant women with a mean age of 28.5 ± 9.4 years, of whom 1446 provided a blood sample. Data on diabetes and pre-diabetes were available from 1407 women, data on inflammation from 1401, data on obesity from *1651* and data on hypertension from *1685*. More details on the survey population is presented elsewhere^16^. The co-existence of obesity, hypertension, elevated HbA1c and inflammation in the same woman is shown in Fig. 1. In all, 70.1% (95% CI 65.7, 74.2) of women suffered from at least one of the investigated cardiometabolic risk factors. Presence of only one risk factor was found in 43.3% (95% CI 38.8, 48.0) of women, with one-third of women found with elevated HbA1c. Co-existence of two of the risk factors was found in 17.1% (95% CI 14.5, 20.0) of women, whereas 7.9% (95% CI 6.3, 9.8) were affected by three risk factors, and 1.8% (95% CI 1.0, 3.4) of women had all four combined.

The prevalence of women with diabetes, elevated HbA1c, obesity, hypertension and any inflammation as well as the bivariate associations between those risk factors and demographic indicators are presented in Table 1. Candidate predictors retained in the multivariate models included age and household sanitation for diabetes; woman’s education and household sanitation for elevated HbA1c; age, woman’s education, residence, local government authority, and wealth quintile for hypertension; age, local government authority, and wealth quintile for obesity, and age for any inflammation.

**Table 1:** Associations between cardio metabolic risk factors and demographic indicators in non-pregnant women, The Gambia, 2018.

Characteristic	Diabetes^a^		Elevated HbA1c^a^		Hypertension^b^		Obesity^c^		Any Inflammation^h^	
	*(N* = *1407)*		*(N* = *1407)*		*(N* = *1685)*		*(N* = *1651)*		*(N* = *1401)*	
	%^d^	(95% CI)^e^	%^d^	(95% CI)^e^	%^d^	%^d^	%^d^	(95% CI)^e^	%^d^	(95% CI)^e^
**Age in years** *(p-value*^*f*^*)*	< *0.05*		*0.40*		< *0.0001*		< *0.0005*		< *0.0001*	
15–19	4.4	(2.3, 8.1)	50.4	(42.4, 58.5)	9.5	(6.6, 13.5)	2.1	(0.7, 6.3)	7.2	(4.7, 11.0)
20–24	7.9	(4.9, 12.5)	52.1	(43.4, 60.6)	12.2	(5.7, 24.3)	4.1	(1.8, 9.3)	14.5	(10.8, 19.1)
25–29	8.8	(4.5, 16.8)	51.0	(39.1, 62.9)	13.1	(8.7, 19.2)	4.7	(2.3, 9.3)	20.6	(12.6, 31.9)
30–34	5.7	(3.0, 10.5)	53.1	(40.5, 65.4)	21.0	(14.7, 29)	16.1	(9.0, 26.9)	22.8	(15.6, 32.1)
35–39	12.5	(6.8, 22.0)	56.7	(43.3, 69.1)	28.3	(17.9, 41.7)	15.1	(10.4, 21.6)	35.0	(28.8, 41.8)
40–44	5.2	(2.0, 12.8)	63.9	(52.9, 73.6)	44.7	(29.4, 61)	32.2	(24.7, 40.8)	45.8	(35.1, 57.0)
45–49	18.9	(8.8, 36.0)	65.2	(41.4, 83.3)	53.8	(40.5, 66.5)	2.1	(0.7, 6.3)	45.3	(32.8, 58.5)
**Woman’s education** *(p-value*^*f*^*)*	*0.21*		< *0.05*		< *0.005*		*0.50*		*0.14*	
Never at school	11.4	(5.5, 22.0)	62.1	(54.6, 69.0)	29.1	(22.5, 36.7)	12.2	(9.0, 16.4)	26.4	(20.9, 32.6)
Primary school	8.1	(4.7, 13.5)	59.7	(50.9, 68.0)	19.4	(14.9, 24.9)	10.7	(7.4, 15.4)	22.0	(14.4, 32.2)
Lower Secondary	5.2	(2.7, 9.6)	49.8	(39.6, 59.9)	16.7	(9.6, 27.4)	12.7	(7.3, 21.4)	18.8	(11.9, 28.5)
Upper Secondary	3.4	(1.3, 8.7)	35.7	(23.8, 49.7)	10.0	(6.5, 15.2)	6.3	(3.5, 11.1)	15.8	(11.6, 21.1)
Higher	7.7	(2.6, 20.5)	53.5	(29.3, 76.2)	11.0	(5.3, 21.5)	10.8	(4.3, 24.4)	32.5	(21.7, 45.5)
**Residence** *(p-value*^*f*^*)*	*0.22*		*0.58*		*0.06*		*0.15*		*0.18*	
Urban	7.2	(4.2, 12.0)	53.6	(44.0, 63.0)	18.8	(15.6, 22.6)	12.3	(9.6, 15.6)	23.9	(21.8, 26.2)
Rural	10.3	(7.7, 13.7)	56.6	(51.4, 61.7)	25.5	(19.8, 32.1)	8.3	(5.2, 13.0)	20.2	(15.9, 25.3)
**Local Gov’t Authority** *(p-value*^*f*^*)*	*0.14*		*0.18*		< *0.005*		*0.09*		*0.23*	
Banjul	13.6	(7.4, 23.8)	57.2	(47.1, 66.7)	28.0	(22, 34.9)	17.1	(12.3, 23.2)	28.8	(23.3, 35)
Kanifing	7.8	(4.8, 12.4)	45.1	(34.5, 56.1)	11.5	(8.1, 16.1)	15.8	(11.6, 21.2)	26.7	(22.6, 31.2)
Brikama	7.1	(3.1, 15.8)	57.7	(43.2, 71.1)	20.0	(15.5, 25.3)	11.4	(7.8, 16.4)	21.4	(18.6, 24.6)
Mansakonko	2.8	(1.5, 5.3)	41.2	(31.3, 51.7)	24.3	(20.5, 28.6)	5.1	(3.8, 7.0)	24.5	(17.1, 33.7)
Kuntaur	19.8	(11.6, 31.8)	67.6	(45.7, 83.8)	12.6	(7.6, 20)	2.7	(1.0, 7.3)	14.8	(7.5, 26.9)
Kerewan	12.9	(7.7, 20.8)	63.3	(54.4, 71.3)	31.4	(18.1, 48.7)	9.6	(5.8, 15.4)	28.3	(18.7, 40.3)
Janjanbureh	8.9	(4.1, 18.5)	56.8	(49.9, 63.4)	18.6	(14, 24.3)	6.8	(4.2, 10.9)	21.6	(16, 28.4)
Basse	5.4	(2.9, 10.0)	49.9	(42.2, 57.6)	29.2	(21.7, 38.1)	10.6	(5.4, 19.9)	20.1	(13.7, 28.5)
**Wealth quintile** *(p-value*^*f*^*)*	*0.57*		*0.21*		*0.08*		< *0.005*		*0.56*	
Poorest	11.9	(7.6, 18.4)	62.2	(54.5, 69.3)	23.6	(17.3, 31.4)	3.9	(2.5, 5.9)	18.8	(13.1, 26.2)
Second	7.8	(4.1, 14.5)	57.4	(43.8, 69.9)	19.9	(13.7, 28)	8.9	(6.7, 11.7)	21.8	(15.4, 29.8)
Middle	7.5	(2.8, 18.6)	60.1	(47.7, 71.3)	25.5	(19.7, 32.4)	13.0	(8.6, 19.2)	24.1	(17.5, 32.2)
Fourth	5.9	(3.3, 10.4)	54.9	(41.6, 67.5)	16.9	(13.2, 21.4)	12.2	(7.9, 18.3)	21.7	(16.6, 27.8)
Wealthiest	7.6	(4.3, 12.9)	39.3	(20.9, 61.4)	16.2	(12.4, 21)	15.7	(10.3, 23.3)	26.5	(21.4, 32.3)
**Household sanitation **^**g**^ *(p-value*^*f*^*)*	< *0.01*		*0.09*		*0.86*		*0.21*		*0.14*	
Inadequate	10.5	(7.1, 15.1)	59.8	(55.0, 64.5)	21.0	(16.5, 26.4)	10.0	(7.5, 13.2)	20.5	(17.4, 24)
Adequate	5.3	(3.2, 8.5)	48.2	(35.7, 60.9)	20.5	(16.7, 24.9)	12.4	(9.1, 16.7)	25.6	(21.2, 30.7)
**Total**	8.1	(5.7, 11.3)	54.5	(47.4, 61.4)	20.9	(17.9, 34.3)	11.4	(9.3, 14.0)	22.9	(20.9, 25.0)

Note: All N’s reflect the total number of subjects with valid data for each condition. N’s for each sub-group analysis vary slightly.

^a^Diabetes defined as glycated hemoglobin (HbA1c) ≥ 6.5% and elevated HbA1c as ≥ 5.7%

^b^Hypertension defined as systolic blood pressure of ≥ 140 mmHg and/or a diastolic blood pressure of ≥ 90 mmHg.

^c^Obesity defined as body mass index (BMI) of ≥ 30 kg/ m2.

^d^Percentages weighted for non-response and survey design.

^e^CI = confidence interval, adjusted for cluster and stratified sampling design.

^f^Chi-square p-value < 0.05 indicates that the proportion in at least one subgroup is statistically significant.

^g^Composite variable of toilet type and if toilet facilities are shared with non-household members; Adequate Sanitation = flush or pour flush toilet or pit latrine with slab not shared with another household; Inadequate sanitation = open pit, bucket latrine, hanging toilet/latrine, no facility, bush, field.

^h^Any inflammation defined as CRP > 3 mg/L and/or AGP > 1 g/L.

Table 2 presents the bivariate associations between the risk factors and nutrition and health indicators in the non-pregnant women. As iron deficiency anemia (IDA) is a composite variable of anemia and iron deficiency (ID), multivariate models excluded IDA if either anemia and/or ID were already included. The following indicators were included as potential predictors into the multivariate model: nutritional status and minimum dietary diversity for diabetes; nutritional status, anemia, ID, and physical activity for elevated HbA1c; nutritional status and physical activity for hypertension; minimum dietary diversity and ID for obesity; and nutritional status, IDA and minimum dietary diversity for any inflammation.

**Table 2:** Associations between cardio metabolic risk factors and nutrition and health indicators in non-pregnant women, The Gambia, 2018.

Characteristic	Diabetes^a^		Elevated HbA1c^a^		Hypertension^b^		Obesity^c^		Any Inflammation^k^	
	%^d^	(95% CI)^e^	%^d^	(95% CI)^e^	%^d^	(95% CI)^e^	%^d^	(95% CI)^e^	%^d^	(95% CI)^e^
**Nutritional status **^**c**^ *(p-value*^*f*^*)*	< *0.005*		< *0.05*		< *0.0001*				< *0.0001*	
Underweight	11.0	(5.4, 20.9)	60.7	(51.8, 68.9)	9.7	(6.1, 15.1)			11.8	(7.3, 18.6)
Normal weight	5.2	(3.3, 7.9)	46.8	(37.2, 56.7)	14.4	(10.6, 19.2)			12.1	(9.1, 15.9)
Overweight	8.3	(4.9, 13.5)	68.0	(52.4, 80.4)	25.5	(18.6, 33.9)			39.1	(29.7, 49.4)
Obesity	15.7	(8.7, 26.5)	60.2	(46.2, 72.7)	39.7	(28.8, 51.7)			60.2	(50.7, 69.1)
**Minimum dietary diversity **^**g**^ *(p-value*^*f*^*)*	*0.09*		*0.51*		*0.12*		< *0.05*		< *0.05*	
Yes	8.7	(6.1, 12.3)	55.2	(47.4, 62.8)	21.9	(18.2, 26.2)	13.2	(10.6, 16.4)	25.3	(22.5, 28.4)
No	6.7	(4.3, 10.1)	52.6	(44.2, 60.9)	18.0	(14.3, 22.2)	7.1	(3.8, 12.7)	17.8	(13.9, 22.5)
**Anemia **^**h**^ *(p-value*^*f*^*)*	*0.34*		< *0.005*		*0.92*		*0.70*		*0.37*	
Yes	8.9	(5.2, 14.8)	59.2	(51.0, 66.8)	19.4	(14.7, 25.2)	11.4	(7.6, 16.8)	21.0	(17.5, 25.0)
No	6.7	(4.6, 9.6)	48.9	(41.8, 56.0)	19.7	(17.0, 22.7)	12.6	(9.8, 16.1)	24.1	(20.2, 28.4)
**Iron deficiency **^**I**^ *(p-value*^*f*^*)*	*0.13*		< *0.05*		*0.47*		*0.07*		*0.44*	
Yes	8.9	(5.7, 13.6)	58.4	(49.9, 66.4)	20.7	(15.8, 26.5)	8.7	(5.5, 13.3)	21.8	(18.5, 25.5)
No	6.9	(4.7, 9.8)	51.5	(44.7, 58.2)	18.8	(15.5, 22.6)	14.6	(10.9, 19.3)	23.7	(21.0, 26.6)
**Iron deficiency anemia **^**I**^ *(p-value*^*f*^*)*	*0.20*		< *0.005*		*0.86*		*0.23*		*0.08*	
Yes	9.8	(5.0, 18.4)	64.4	(54.0, 73.7)	20.1	(13.3, 29.0)	8.6	(4.5, 15.9)	18.4	(14.1, 23.7)
No	6.8	(5.0, 9.2)	50.0	(43.3, 56.7)	19.3	(16.3, 22.8)	12.9	(10.4, 15.9)	24.2	(21.6, 26.9)
**Vitamin A insufficiency **^**j**^ *(p-value*^*f*^*)*	*0.93*		*0.71*		*0.50*		*0.14*		*0.99*	
Yes	7.5	(5.0, 11.2)	55.7	(48.9, 62.3)	22.0	(14.4, 32.3)	8.3	(4.3, 15.5)	22.9	(16.7, 30.6)
No	7.8	(4.8, 12.3)	53.9	(45.8, 61.8)	18.9	(15.4, 22.9)	13.2	(10.9, 15.9)	22.9	(20.1, 25.9)
**Physically active** *(p-value*^*f*^*)*	*0.95*		*0.06*		*0.09*		*0.87*		*0.80*	
Yes	8.3	(3.3, 19.0)	64.0	(55.0, 72.0)	15.8	(10.9, 22.2)	12.1	(6.0, 22.7)	21.8	(14.5, 31.3)
No	8.1	(5.6, 11.5)	53.3	(45.5, 60.9)	21.2	(18.2, 24.5)	11.4	(9.0, 14.3)	23.0	(20.7, 25.5)

^a^Diabetes defined as glycated hemoglobin (HbA1c) ≥ 6.5% and elevated HbA1c as ≥ 5.7%

^b^Hypertension defined as systolic blood pressure of ≥ 140 mmHg and/or a diastolic blood pressure of ≥ 90 mmHg.

^c^Underweight, overweight, and obesity defined as body mass index (BMI) of < 18.5 kg/ m2, ≥ 25- < 30 kg/ m2 and ≥ 30 kg/ m2, respectively.

^d^Percentages weighted for non-response and survey design.

^e^CI = confidence interval, adjusted for cluster and stratified sampling design.

^f^Chi-square p-value < 0.05 indicates that the proportion in at least one subgroup is statistically significantly.

^g^Minimum dietary diversity was met if the number of food groups consumed the day prior to the interview was ≥ 5 (25).

^h^Anemia defined as hemoglobin < 120 mg/L.

^I^Iron deficiency defined as serum ferritin < 15 µg/l; iron deficiency anemia defined as low serum ferritin and low hemoglobin.

^j^Vitamin A insufficiency defined as retinol binding protein < 1.05 μmol/L.

^k^Any inflammation defined as CRP > 3 mg/L and/or AGP > 1 g/L.

Table 3 presents the bivariate associations between the different risk factors. Significant associations were found between obesity and diabetes, obesity and hypertension, and obesity and inflammation, but not between obesity and elevated HbA1c. Inflammation was significantly associated with diabetes, hypertension, and obesity; the association between inflammation and elevated HbA1c was close to significant.

**Table 3:** Associations between different cardio metabolic risk factors in non-pregnant women, The Gambia, 2018.

Characteristic	Diabetes^a^		Elevated HbA1c^a^		Hypertension^b^		Obesity^c^		Any Inflammation^k^	
	*(N* = *1407)*		*(N* = *1407)*		*(N* = *1685)*		*(N* = *1651)*		*(N* = *1378)*	
	%^d^	(95% CI)^e^	%^d^	(95% CI)^e^	%^d^	(95% CI)^e^	%^d^	(95% CI)^e^	%^d^	(95% CI)^e^
**Diabetes**^**a**^ *(p-value*^*f*^*)*					*0.73*		< *0.005*		< *0.05*	
Yes					20.4	(14.3, 28.3)	25.0	(15.9, 37.0)	34.2	(22.5, 48.1)
No					18.9	(15.4, 22.9)	11.4	(8.7, 14.6)	21.9	(20, 24)
**Elevated HbA1c**^**a**^ *(p-value*^*f*^*)*					*0.89*		*0.24*		*0.08*	
Yes					19.2	(15.0, 24.1)	13.8	(11.0, 17.3)	25.1	(21.6, 28.9)
No					18.8	(15.1, 23.2)	10.9	(7.2, 16.0)	20.2	(17.4, 23.4)
**Hypertension**^**b**^ *(p-value*^*f*^*)*	*0.73*		*0.89*				< *0.0001*		< *0.0001*	
Yes	8.4	(4.3, 15.9)	54.8	(44.3, 64.9)			24.6	(16.4, 35.2)	38.7	(32.5, 45.3)
No	7.7	(5.6, 10.6)	54.2	(47.0, 44.3)			8.5	(6.7, 10.9)	19.1	(16.5, 21.9)
**Obesity**^**c**^ *(p-value*^*f*^*)*	< *0.005*		*0.24*		< *0.0001*				< *0.0001*	
Yes	15.7	(8.7, 26.5)	60.2	(46.2, 72.6)	39.7	(28.8, 51.8)			60.2	(50.7, 69.1)
No	6.7	(4.6, 9.7)	53.5	(46.6, 60.3)	15.8	(12.6, 19.7)			17.7	(15.5, 20.1)
**Inflammation**^**k**^ *(p-value*^*f*^*)*	< *0.05*		< *0.08*		< *0.0001*		< *0.0001*			
Elevate CRP and/or AGP	11.5	(7.4, 17.4)	59.6	(49.5, 68.9)	32.9	(25.9, 40.9)	31.9	(25.5, 39.1)		
No inflammation	6.6	(4.1, 10.2)	52.7	(46.2, 59.1)	15.5	(12.4, 19.2)	6.2	(4.3, 8.8)		

^a^Diabetes defined as glycated hemoglobin (HbA1c) ≥ 6.5% and elevated HbA1c as HbA1c ≥ 5.7%

^b^Hypertension defined as systolic blood pressure of ≥ 140 mmHg and/or a diastolic blood pressure of ≥ 90 mmHg

^c^Obesity defined as body mass index (BMI) of ≥ 30 kg/m^2^, respectively

^d^Percentages weighted for non-response and survey design

^e^CI = confidence interval, adjusted for cluster and stratified sampling design

^f^Chi-square p-value < 0.05 indicates that the proportion between the subgroups is statistically significantly

^k^Any inflammation defined as CRP > 3 mg/L and/or AGP > 1 g/L

When looking at AGP and CRP separately, HbA1c was significantly associated with AGP, but not CRP. Overall, 68.6% of women with high AGP had elevated HbA1c, but only 52.7% of women with normal AGP had elevated HbA1c (p < 0.001); whereas 58.5% of women with elevated CRP had high HbA1c compared to 53.2% of women with normal CRP (p = 0.27). The prevalence of hypertension was higher in women with elevated CRP (33.9% vs. 15.8%, p < 0.0001) and in women with elevated AGP, albeit without statistical significance (30.9% vs. 18.2%, p = 0.08). Moreover, the prevalence of elevated AGP (26.1% vs. 7.9%, p < 0.0001) and CRP (58.5% vs. 15.3%, p < 0.0001) was higher in obese women compared to women without obesity.

Multivariate analyses (Table 4) showed that overweight and obese women had a higher risk to develop diabetes compared to the normal weight. Further, living in households with inadequate sanitation increased the risk for diabetes compared to living in households with adequate sanitation.

**Table 4:** Adjusted relative risk of diabetes, hypertension, obesity in non-pregnant women, The Gambia, 2018.

Model and characteristic	Category	Adjusted relative risk^a^	95% CI	P-value
**Diabetes model (n = 1,402)**				
Nutritional status	Underweight	1.39	(0.87, 2.22)	0.170
	Normal	Referent		
	Overweight	1.97	(1.31, 2.95)	< 0.01
	Obese	2.40	(1.56, 3.71)	< 0.001
Sanitation	Adequate	Referent		
	Not adequate	1.67	(1.17, 2.34)	< 0.01
**Elevated HbA1c model (n = 1,358)**				
Educational attendance	Never attended school	Referent		
	Primary school	0.95	(0.82, 1.09)	0.435
	Lower Secondary School	0.83	(0.72, 0.96)	< 0.05
	Upper Secondary School	0.84	(0.72, 0.98)	< 0.05
	Higher	0.94	(0.76, 1.16)	0.560
Nutritional Status	Underweight	1.11	(0.97, 1.28)	0.135
	Normal	Referent		
	Overweight	1.21	(1.07, 1.38)	< 0.01
	Obese	1.31	(1.14, 1.51)	< 0.001
Iron deficiency	No	Referent		
	Yes	1.20	(1.09, 1.33)	< 0.001
**Hypertension model (n = 1,358)**				
Age Group (years)	15–19	Referent		
	20–24	1.17	(0.74, 1.84)	0.502
	25–29	1.20	(0.75, 1.92)	0.435
	30–34	1.79	(1.16, 2.76)	< 0.01
	35–39	2.13	(1.40, 3.23)	< 0.001
	40–44	2.70	(1.76, 4.14)	< 0.001
	45–49	2.91	(1.90, 4.44)	< 0.001
Educational attendance	Never attended school	Referent		
	Primary school	0.89	(0.67, 1.19)	0.426
	Lower Secondary School	0.83	(0.62, 1.11)	0.219
	Upper Secondary School	0.64	(0.44, 0.94)	0.022
	Higher	0.58	(0.34, 0.99)	< 0.05
Inflammation	No	Referent		
	Yes	1.42	(1.14, 1.78)	< 0.01
Nutritional Status	Underweight	1.07	(0.75, 1.52)	0.707
	Normal	Referent		
	Overweight	1.22	(0.93, 1.60)	0.151
	Obese	1.84	(1.40, 2.41)	< 0.001
**Obesity model (n = 1,640)**				
Age Group (years)	15–19	Referent		
	20–24	2.33	(0.99, 5.47)	0.052
	25–29	2.94	(1.28, 6.79)	< 0.05
	30–34	10.36	(4.96, 21.64)	< 0.001
	35–39	7.99	(3.78, 16.90)	< 0.001
	40–44	13.64	(6.45, 28.84)	< 0.001
	45–49	10.86	(5.04, 23.40)	< 0.001
Wealth quintile	Lowest	Referent		
	Second	1.82	(1.03, 3.21)	< 0.05
	Middle	2.33	(1.36, 4.01)	< 0.01
	Fourth	3.72	(2.17, 6.35)	< 0.001
	Highest	3.93	(2.39, 6.48)	< 0.001
**Inflammation model (n = 1,383)**				
Age group (years)	15–19	Referent		
	20–24	1.05	(0.70, 1.56)	0.815
	25–29	1.22	(0.83, 1.81)	0.312
	30–34	1.46	(1.02, 2.09)	< 0.05
	35–39	1.87	(1.31, 2.66)	< 0.01
	40–44	1.86	(1.28, 2.71)	< 0.01
	45–49	1.95	(1.32, 2.89)	< 0.01
Nutrition status	Underweight	0.94	(0.64, 1.37)	0.735
	Normal	Referent		
	Overweight	2.03	(1.59, 2.60)	< 0.001
	Obese	3.28	(2.60, 4.15)	< 0.001

^a^Calculated using relative risk from Poisson regression.

Overweight and obesity also increased the risk for elevated HbA1c compared to normal weight. Further, women with ID had an increased risk for elevated HbA1c compared to those without. On the other hand, attending secondary school slightly reduced the risk of elevated HbA1c.

Increasing age as well as increasing weight resulted in higher risks for hypertension. Also, women with inflammation had a higher risk to develop hypertension compared to those without. Contrary, the risk for hypertension continuously decreases with increasing levels of education.

The risk for obesity strongly increased with increasing age and household wealth.

For inflammation, the risk was higher in women with overweight and obesity compared to normal weight and for older women compared to young ones.

## Discussion

Our study found that more than 2 out of 3 Gambian women had at least one of the investigated cardiometabolic risk factors. This finding is concerning and suggests that NCDs are widespread in The Gambia and that policies and programs to address NCDs should be further developed and accelerated. As part of its 2013–2020 Global NCD Action Plan, WHO aims to reduce hypertension as well as diabetes and obesity^3^. The current WHO national NCD targets for The Gambia are limited to a reduction in tobacco and alcohol use, and no targets have been formulated for hypertension, obesity and diabetes ^17^. Our results demonstrate that The Gambia should strongly consider revising its NCD targets as NCDs are common despite smoking and alcohol consumption being very uncommon^18^.

### Obesity

One potential entry point to reducing NCDs in The Gambia is through obesity reduction. Although still relatively low in The Gambia^16^, the prevalence of obesity increased by more than 50% in 5 years, from 7.3% in 2013^19^ to 11.1% in 2018^20^. Despite the relatively low obesity prevalence, our analysis found repeated and significant associations between obesity and the other investigated cardiometabolic risk factors, indicating that obesity is key to the development of other metabolic disorders in The Gambia. The direction of the association between obesity and other investigated factors has been well documented^21,22^  : in obese people, the secretion of several adipokines and cytokines, which are involved in various metabolic processes including carbohydrate and lipid metabolism compounds is increased and often associated with low grade inflammation and the development of metabolic disorders such as type 2 diabetes and certain CVD^23,24^. Further, according to the International Diabetes Federation (IDF), obesity is playing a central role in the development of the metabolic syndrome^25^, a clustering of related metabolic disturbances which have been shown to increase the risk of CVD and type 2 diabetes, such as prediabetes, hypertension and visceral obesity^26,27^. Thus, we can presume that if obesity is reduced, cases of elevated HbA1c, diabetes, hypertension, and inflammation will also be reduced.

### Diabetes and pre-diabetes

The last estimate from 1997 reported less than 1% of the adult population of The Gambia being diabetic^28^, which is a mere fraction of what was found in 2018. The high prevalence of diabetes and also the large proportion of individuals who exhibit prediabetes are of major concern, particularly since prediabetic individuals have a substantially increased risk to develop diabetes^29^. Furthermore, a series of subclinical abnormalities, such as increased blood levels of low-density lipoprotein cholesterol or triglycerides, as well as obesity and hypertension are often present in prediabetic patients^30^. Meta-analyses found that prediabetes also favors the development of CVD^31,32^, although it is unclear if this effect is caused by the aforementioned abnormalities or prediabetes itself^33^.

A strong increase in diabetes prevalence can be observed in SSA in general^34^ and in Western Africa in particular^35^. The 2019 Diabetes Atlas published by the International Diabetes Federation estimates that the diabetes prevalence in SSA is about 4%, with country-specific prevalence rates ranging from 2.1 to 7.1% (10). These estimates are slightly lower than the estimates from our study. However, the current true prevalence of diabetes in Gambian adults is uncertain, as our study did not include older women, who have a higher prevalence of diabetes than younger women^36^, and it did not include men, who may have a lower prevalence of diabetes than women^37^. One potential reason for the slightly higher diabetes prevalence found in The Gambia compared to those reported from other SSA countries could be the use of HbA1c as a biomarker for diabetes. ID and IDA can cause elevation in glycated hemoglobin by altering the erythrocyte turnover and lead to an over-estimation of the prevalence of prediabetes and diabetes^38^. We find the prevalence of diabetes in women with ID 2 percentage points, and with IDA 3 percentage points higher—albeit not significantly—than in iron replete women. Yet, the prevalence of elevated HbA1c was significantly higher among women with ID and IDA compared to women without.

Although we found that obesity was significantly associated with an increased risk of elevated HbA1c and diabetes, about 55% of diabetic women in our study had low to normal BMIs. This finding is similar to other studies, that found that a larger proportion of diabetic persons in SSA have low to normal BMI compared to diabetic persons in Europe and North America, which might be related to the high prevalence of diabetes in SSA due to impaired insulin secretion rather than insulin resistance as it is the case in Europe and North America^39^.

Further, our results indicate that inadequate sanitation was associated with diabetes. Prolonged exposure to inadequate sanitation can cause environmental enteropathy^40^, which has been associated with the development of diabetes via inflammatory and non-inflammatory pathways^41,42^.

### Inflammation

We found that inflammation was associated with a higher prevalence of obesity, hypertension and diabetes. Although not being part of the metabolic syndrome, inflammation is recognized as a central mechanism underlying its pathophysiology^27^. There is growing evidence that chronic low-grade inflammation favors the development of insulin resistance and type 2 diabetes^43–45^. On the other hand, diabetes often leads to a dysfunctional immune system, with impaired immune response, rendering the individual more vulnerable to infection. Moreover, hyperglycemic states in diabetic and non-diabetic persons can negatively alter the outcome of infections^46,47^. Consequently, diabetes is often associated with infectious diseases such as pneumonia, tuberculosis, HIV and particularly infections originating in the urinary tract^48^ and increases the risk for morbidity and mortality from various infectious diseases^49^. Thus, an increasing prevalence of diabetes might hamper the treatment and control of certain infectious diseases in The Gambia^50^. Moreover, human and animal studies indicate that inflammation causes hypertension^51^.

### Hypertension

According to the WHO, with an estimated 46% of adults older than 25 years of age affected, the prevalence of hypertension is highest in the African region; however, it is estimated that many cases remain undiagnosed because of weak health systems. A STEPwise WHO survey in 2010 in The Gambia estimated that 26.1% of women 25–64 years of age were hypertensive, 71% of whom were undiagnosed^18^. This estimate is higher than that found in this study, but this difference may be explained by the different age ranges of the two surveys. In 2010 the prevalence of hypertension was 16.9% and 33.6% in women aged 25–34 years and 35–44 years, respectively, compared to 16.6% and 35.0% in 2018.

### Limitations

Our study contains notable limitations. First, the cross-sectional nature of the data precludes any inference of causality. Second, as many of the cardiometabolic risk factors we investigated are interrelated, we generally do not imply the direction of the associations we observe^26,52^. The sole exception are the well documented associations between obesity and other factors as noted above. Third, although WHO recommends the use of HbA1c to measure diabetes^53^, we acknowledge that the high prevalence of anemia and/ or ID in Gambian women may have resulted in a small overestimation of the prevalence of diabetes and elevated HbA1c. Moreover, HbA1c thresholds vary by ethnic group; persons of African and Asian descent tend to have higher HbA1c levels compared to Caucasians^54–56^. Hence, more research on HbA1c as a biomarker in different settings and the establishment of ethnic-specific cut-offs defining prediabetes and diabetes is warranted.

## Conclusion

The high prevalence of cardiometabolic risk factors in non-pregnant women of reproductive age in The Gambia is of concern, particularly since a large proportion of women are affected by more than one risk factor. While obesity was identified as a risk factor of diabetes, elevated HbA1c, hypertension, and inflammation, large proportions of the non-obese women also exhibited elevated HbA1c and/or hypertension and/or inflammation. Hence, more research on drivers of cardiometabolic risk factors is warranted to formulate multiple strategies for their containment.

## Methods

### Survey design and participants

The Gambia National Micronutrient Survey was conducted between 13th March and 4th May 2018. This was a nationwide cross-sectional stratified survey based on a probability sample to produce estimates with acceptable precision for priority indicators of nutritional status and nutrition-related non-communicable diseases in non-pregnant women of reproductive age. The sample was drawn separately from the urban and rural areas in each of the eight Local Government Areas, resulting in 14 strata (the local government areas of Banjul and Kanifing had no rural strata because they are entirely urban). A two-stage sampling procedure was used. The list of census enumeration areas selected as primary sampling units for the 2018 Multi Indicator Cluster Survey (MICS) served as sampling frame for the first stage of sampling. At this first stage of sampling for The Gambia National Micronutrient Survey 2018, MICS-selected primary sampling units within each stratum were randomly selected with equal probability from the 390 enumeration areas enrolled in the MICS. In the 2nd stage of sampling, because household sizes vary by region, different numbers of households, which already participated in the MICS, were selected within each selected primary sampling unit^16^; selection of households within a primary sampling unit were selected using simple random sampling from updated household lists. All non-pregnant women 15–49 years of age, pregnant women any age and children 0–59 months of age living in the selected households were included in the survey. All non-pregnant women who participated in the Gambia National Micronutrient survey and with data on at least one of the investigated cardiometabolic risk factor were included in this study.

### Data collection

Prior to data collection, experienced field workers received intense training as described elsewhere^16^. First, oral consent was obtained and a short household questionnaire was administered to the head of the household. After obtaining written informed consent from each recruited woman, short questionnaires were administered. Anthropometric measurements were done using a medical scale (SECA, Hamburg, Germany) and a standard wooden height board (UNICEF, Copenhagen, Denmark); and blood was collected as previously described^16^.

### Diagnostic tests and clinical thresholds

The study assessed the following cardiometabolic risk factors: Pre-diabetes, diabetes, hypertension, obesity and inflammation.

HbA1c concentration was used to assess the presence of diabetes and pre-diabetes. Diabetes was defined as HbA1c ≥ 6.5% and pre-diabetes as HbA1c ≥ 5.7% to < 6.5%, whereas elevated HbA1c was defined as ≥ 5.7%^57^. HbA1c was measured by using the Hemocue® HbA1c 501 (HemoCue, Ångelholm, Sweden). While this device was developed as a point-of-care device, we used it in field settings by powering the device with rechargeable battery packs. Quality control of the device was done using the “daily” and “monthly” check cartridges delivered with the device. In addition, daily quality control was done using control blood furnished by the instrument manufacturer which had two different HbA1c concentrations (range 7.1–8.3% and range 8.8–10.2%). Control blood was kept refrigerated at 2–8 °C for the duration of the field work to prevent degradation.

Hypertension was defined as systolic blood pressure of ≥ 140 mmHg and/or a diastolic blood pressure of ≥ 90 mmHg^58^. Blood pressure was measured once on the left arm of each woman while the woman was sitting using an automatic blood pressure monitor (OMRON M300, Kyoto, Japan).

Chronic energy deficiency and overnutrition were assessed using the body mass index (BMI; kg/m^2^). Cut-off points for BMI were as follows: < 16.0 severe chronic energy deficiency; 16.0–16.9 moderate chronic energy deficiency; 17.0–18.4 at-risk for energy deficiency; 18.5–24.9 normal; 25.0–29.9 overweight; ≥ 30.0 obese ^59^*.*

Inflammation was defined as AGP > 1 g/L^60^ or CRP > 3 mg/L^61^. Using the aforementioned cut-off values, each individual was assigned to one of the following four inflammation categories: elevated CRP only, elevated CRP and AGP, elevated AGP only, and no inflammation.

Women were considered anemic if hemoglobin concentrations were < 120 g/L^66^. ID and vitamin A insufficiency were defined as ferritin concentrations < 15 µg/L^62^ and RBP concentrations < 1.05 µmol/L^63^, respectively. Ferritin concentrations were adjusted for inflammation using CRP and AGP^64^.

Hemoglobin concentrations were measured on-site using a portable haemoglobinometer (Hb 301 + Analyser, HemoCue® AB, Ängelholm, Sweden). Daily quality control was done using control blood furnished by the instrument manufacturer (low, medium and high concentration).

Capillary blood samples (400–500 µl) were collected from a fingerprint into Microvette® tubes, and kept between 4 and 8 °C until they were centrifuged, after which serum was aliquoted and frozen at − 20 °C. Serum samples were subsequently stored in Banjul at − 280 °C and were shipped to the VitMin Laboratory in Germany for analysis of concentrations of C-reactive protein (CRP), alpha-1-acid glycoprotein (AGP), ferritin, and retinol binding protein (RBP) using the sandwich ELISA approach^65^.

### Data management and statistical analysis

Data were collected in the field using tablet computers and entered into an application built using CSPro version 7, with rigorous consistency checks after collection. Data analysis was done using Stata/IC version 14.2 (STATACorp, College Station, Texas, USA). Statistical weights were applied to all data to account for the unequal probability of selection in the 14 strata. For categorical variables, proportions were calculated to derive the prevalence of the different outcomes. The statistical precisions of prevalence estimates were assessed by using 95% confidence intervals. All measures of precision, including confidence limits and chi square p values for differences, were calculated accounting for the complex cluster and stratified sampling used in the survey^16^.

Descriptive statistics were calculated across all strata together, for each stratum separately, and by age group. Factors associated with obesity, hypertension, elevated HbA1c, diabetes mellitus, and inflammation were identified using bivariate analyses. Variables with associations at p < 0.1 in the bivariate analyses were included in the respective multivariate models. During the construction of the multivariate models, backwards elimination was used to construct parsimonious models that only contained statistically significant (i.e. p < 0.05) covariates.

The Poisson regression^67^, which yields adjusted risk ratios (aRRs), was considered superior to regression models that yield odds ratios (e.g. logistic regression), as the relatively high prevalence of all outcome variables examined in our analysis violated the rare disease assumption^68^.

### Ethics approval and consent to participate

Ethical approval for the survey (R18014) was obtained from The Gambia Government/MRC Joint Ethics Committee and the School of Medicine and Allied Health Sciences Research & Publication Committee. The survey was conducted in accordance with the approved protocol.

Oral consent was obtained for household interviews from the household head or other knowledgeable household member. Written informed consent was obtained from selected women who were at least 18 years of age. For women younger than 18 years, written informed consent was sought from a parent or legal guardian. If any consenting survey participants were unable to read and write, the consent form was read out loud to them and a fingerprint was taken in lieu of a signature, or the respondent could assign a witness to sign on her behalf. Survey respondents diagnosed with severe anemia and/ or severe acute malnutrition were referred to the local health facility for further diagnosis and treatment.

## Data Availability

The data that support the findings of this study are available from The Gambia's National Nutrition Agency (NaNA), but restrictions apply to the availability of these data, which were used with the permission of NaNA, and so are not publicly available. Data are however available from the authors upon reasonable request and with permission of NaNA.
